# Supplementary figures and images for: A global phylogenetic analysis in order to determine the host species and geography dependent features present in the evolution of avian H9N2 influenza hemagglutinin
Source: PeerJ. 2014 Oct 30;2:e655. doi: 10.7717/peerj.655 (PMC4217197; doi:10.7717/peerj.655)

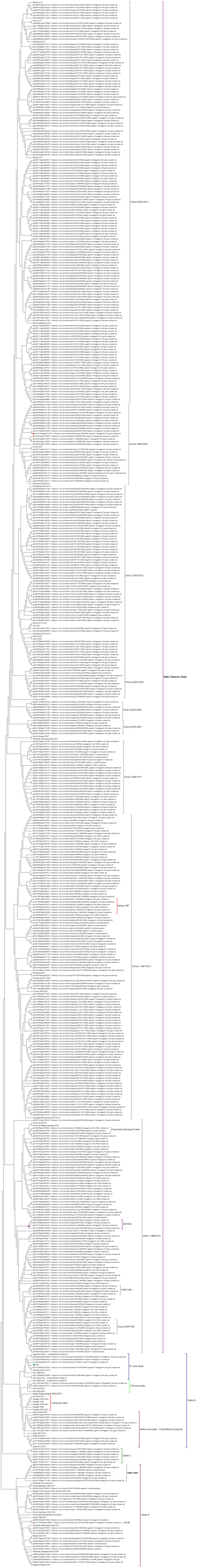

Supplement: Figure S1 — In this figure all of the clades have been expanded to show the leaf nodes. [file peerj-02-655-s001.png]
