# Supplementary material for: A global phylogenetic analysis in order to determine the host species and geography dependent features present in the evolution of avian H9N2 influenza hemagglutinin
Source: PeerJ. 2014 Oct 30;2:e655. doi: 10.7717/peerj.655 (PMC4217197; doi:10.7717/peerj.655)
Supplement: Table S1 — Results are evaluated using Bayesian Information Criteria, Akaike’s Information Criteria and Log Likelihood. [file peerj-02-655-s003.docx]

| Substitution Model | BIC | AICc | Log Likelihood |
| --- | --- | --- | --- |
| JTT+G | 8701.867 | 7504.233 | -3603.21 |
| JTT+G+I | 8711.96 | 7506.246 | -3603.2 |
| JTT+I | 8730.219 | 7532.585 | -3617.39 |
| JTT | 8763.723 | 7574.169 | -3639.19 |
| WAG+G | 8780.574 | 7582.939 | -3642.56 |
| WAG+G+I | 8790.678 | 7584.964 | -3642.56 |
| WAG+I | 8809.263 | 7611.629 | -3656.91 |
| WAG+I | 8809.263 | 7611.629 | -3656.91 |
| Dayhoff+G | 8820.88 | 7623.246 | -3662.72 |
| Dayhoff+G+I | 8830.592 | 7624.878 | -3662.52 |

JTT = Jones Taylor Thornton, WAG = Whelan and Goldman, Dayhoff = PAM/Dayhoff
