# Supplementary material for: A global phylogenetic analysis in order to determine the host species and geography dependent features present in the evolution of avian H9N2 influenza hemagglutinin
Source: PeerJ. 2014 Oct 30;2:e655. doi: 10.7717/peerj.655 (PMC4217197; doi:10.7717/peerj.655)
Supplement: Table S2 — Results are evaluated using Bayesian Information Criteria, Akaike’s Information Criteria and Log Likelihood. [file peerj-02-655-s004.docx]

| Substitution Model | BIC | AICc | Log Likelihood |
| --- | --- | --- | --- |
| TN93+G | 65198.280 | 64090.626 | -31928.169 |
| GTR+G | 65209.540 | 64073.491 | -31916.594 |
| TN93+G+I | 65209.750 | 64092.631 | -31928.169 |
| GTR+G+I | 65221.009 | 64075.496 | -31959.845 |
| HKY+G | 65250.164 | 64151.974 | -31959.845 |
| HKY+G+I | 65261.633 | 64153.979 | -31959.845 |
| TN93 | 65371.568 | 64273.378 | -32020.547 |
| GTR | 65383.034 | 64256.450 | -32009.076 |
| TN93+I | 65383.034 | 64275.386 | -32020.549 |
| GTR+I | 65394.513 | 64258.465 | -32009.081 |
